# The genome of *Bifidobacterium pseudocatenulatum* IPLA 36007, a human intestinal strain with isoflavone-activation activity

**DOI:** 10.1186/1757-4749-6-31

**Published:** 2014-07-23

**Authors:** Ángel Alegría, Susana Delgado, Lucía Guadamuro, Ana Belén Flórez, Giovanna E Felis, Sandra Torriani, Baltasar Mayo

**Affiliations:** 1 Departamento de Microbiología y Bioquímica de Productos Lácteos, Instituto de Productos Lácteos de Asturias (IPLA-CSIC), Carretera de Infiesto, s/n, 33300 Villaviciosa, Asturias, Spain; 2 Dipartimento di Biotecnologie, Università degli Studi di Verona, Strada Le Grazie, 15, 37134 Verona, Italy

## Abstract

**Background:**

*Bifidobacterium* species, including *Bifidobacterium pseudocatenulatum*, are among the dominant microbial populations of the human gastrointestinal tract. They are also major components of many commercial probiotic products. Resident and transient bifidobacteria are thought to have several beneficial health effects. However, our knowledge of how these bacteria interact and communicate with host cells remains poor. This knowledge is essential for scientific support of their purported health benefits and their rational inclusion in functional foods.

**Results:**

This work describes the draft genome sequence of *Bifidobacterium pseudocatenulatum* IPLA 36007, a strain isolated as dominant from the feces of a healthy human. Besides several properties of probiosis, IPLA 36007 exhibited the capability of releasing aglycones from soy isoflavone glycosides. The genome contains 1,851 predicted genes, including 54 genes for tRNAs and fie copies of unique 16S, 23S and 5S rRNA genes. As key attributes of the IPLA 36007 genome we can mention the presence of a lysogenic phage, a cluster encoding type IV fimbriae, and a locus encoding a clustered, regularly interspaced, short, palindromic repeat (CRISPR)-Cas system. Four open reading frames (*orf*s) encoding β-glucosidases belonging to the glycosyl hydrolase family 3, which may act on isoflavone glycosides, were encountered. Additionally, one gene was found to code for a glycosyl hydrolase of family 1 that might also have β-glucosidase activity.

**Conclusion:**

The availability of the *B. pseudocatenulatum* IPLA 36007 genome should allow the enzyme system involved in the release of soy isoflavone aglycones from isoflavone glycosides, and the molecular mechanisms underlying the strain’s probiotic properties, to be more easily understood.

## Introduction

*Bifidobacterium* species are majority bacteria among those inhabiting the gastrointestinal tract (GIT) of animals and humans. They play important roles in maintaining human health via the digestion of foods, production of essential vitamins, and metabolization of endogenous and exogenous compounds, as well as by preventing the colonization and/or overgrowth of pathogens in the GIT [1]. Molecular analyses have shown that members of the *Bifidobacterium catenulatum* group (which includes *B. catenulatum* and *B. pseudocatenulatum*) are abundant in fecal samples from adult humans [2,3]. *B. pseudocatenulatum* strains have a number of probiotic properties, such as the possession of antinutrient-degrading enzymes [4], the ability to bind mutagenic aromatic amines [5], and the capacity to reduce cholesterol levels [6]. However, compared to other bifidobacterial species, the genome of *B. pseudocatenulatum* has been very little explored. The Genomes Online Database (GOLD) (http://www.genomesonline.org) only contains the draft sequence of a single strain, *B. pseudocatenulatum* DSM 20438 (Gi02660), plus recently released incomplete sequences of five other strains (D2CA; TSDC19.1-1.1; TSDC19.1-1.2; TSDC19.1-1.3; and TSDC17.2-1.1). Sequence analysis of additional *B. pseudocatenulatum* strains would provide greater insight into the intra-specific variation of this species, and supply information on the genetics that underlay strain-specific capabilities. Recently, *B. pseudocatenulatum* has been used as a cloning host for the expression of natural [7] and synthetic [8] genes. Genomic analyses of strains of this species might allow the confident use of this bacterium in other biotechnological applications.

Bifidobacterial strains have been shown to be involved in the conversion of isoflavone glycosides into aglycones [9,10], a key step in making isoflavones bioavailable and harnessing their estrogenic activity [11]. Indeed, the genomes of sequenced bifidobacteria show an impressive array of genes coding for glycosyl hydrolases, including β-glucosidases, which are thought to be involved in the release of aglycones from dietary polyphenols such as soy isoflavones [12,13]. However, the enzyme(s) involved in the hydrolysis of soy isoflavone glycosides remain(s) mostly unknown. So far, a β-glucosidase from *Bifidobacterium animalis* subsp. *lactis* has been shown to possess aglycone-releasing activity from isoflavones by cloning and expression of its encoding gene in *Bifidobacterium bifidum*[14].

The present work provides a draft genome sequence for *B. pseudocatenulatum* IPLA 36007, an intestinal human strain able to release aglycones from the soy isoflavone glycosides daidzin and genistin. This capability endows it with properties of interest in terms of its use in functional foods.

## Materials and methods

### Isolation and DNA preparation

*B. pseudocatenulatum* IPLA 36007 was isolated among the dominant bacteria from fecal samples of a healthy human, in a study approved by The Ethic Committee of the Asturias Principality, Spain [15]. The strain was grown anaerobically at 37°C in MRS medium (Merck, Darmstadt, Germany) supplemented with 0.25% cysteine (Merck). Genomic DNA was extracted and purified from pure cultures using the GenElute™ Bacterial Genomic DNA kit (Sigma-Aldrich, St. Louis, Miss., USA) following the manufacturer’s instructions for extracting DNA from Gram-positive bacteria. The concentration and quality of the DNA was measured using an Epoch microvolume spectrophotometer (BioTek Instruments, Winooski, Vt., USA).

### Aglycone releasing-activity from isoflavone glycosides

Strains were incubated anaerobically in a MRS basal medium without dextrose and supplemented with 2% cellobiose and 100 μM daidzin or ginistin (Sigma-Aldrich) at 37°C for 24 h. One ml cultures were centrifuged and the cells suspended in the same volume of 0.1 M sodium acetate buffer pH 4.1. Isoflavones and derivatives were then extracted with ethyl acetate (Sigma-Aldrich). The organic phase was evaporated and the dried pellet suspended in 100 μl of methanol. Five 5 μl were used for analysis by TLC in silica gel 60 F254 plates (Merck). Isoflavones were separated in a toluene:acetone (2:1) solvent system, revealed by UV light at 365 nm in a transilluminator and visualized with an ImageQuant 350 (GE Healthcare Bio-Sciences, Buckinghamshire, UK).

### Genome sequencing, assembly and annotation

A genomic library of 0.5 kbp was constructed and paired-end sequenced (approximately 155-fold coverage) using a HiSeq 1000 System sequencer (Illumina, Inc., San Diego, CA, USA). Quality-filtered reads were assembled in contigs using Velvet software v.1.2.10. (https://www.ebi.ac.uk/~zerbino/velvet/). Gaps within the contigs were closed by direct sequencing of amplicons obtained by PCR with oligonucleotide primers designed to anneal in the flanking regions. The genome was annotated with the RAST annotation system (http://rast.nmpdr.org/) and the NCBI Prokaryotic Genome Annotation Pipeline (http://www.ncbi.nlm.nih.gov/genome/annotation_prok/). The KEGG Pathway (http://www.genome.jp/kegg/pathway.html), Uniprot (http://www.uniprot.org) and COG (http://www.ncbi.nlm.nih.gov/COG) databases were consulted for description of specific genes and proteins. If required, DNA and deduced protein sequences were individually subjected to BLAST analysis (http://blast.ncbi.nlm.nih.gov/Blast.cgi). Multi-blast protein comparisons were performed with the CLC Bioinformatics Database software package (CLC bio, Aarhus, Denmark).

### Nucleotide sequence accession numbers

The results of this Whole Genome Shotgun project have been deposited in the GenBank database under accession number JEOD00000000. The version described in this paper is JEOD01000000.

## Results and discussion

**Figure 1:**
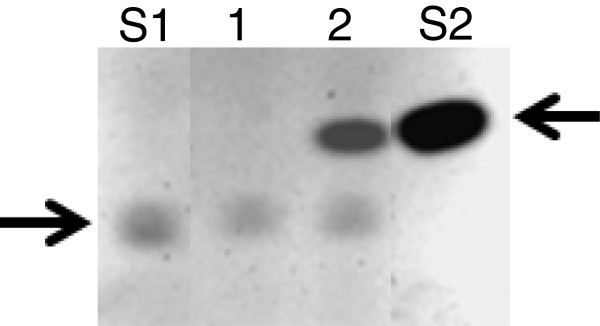
**TLC chromatogram showing the conversion of daidzin to daidzein by *B. pseudocatenulatum *IPLA 36007.** Order: line1, daidzin incubated with whole cells a negative *Bifidobacterium longum* strain; line 2, daidzin incubated with cells of *B. pseudocatenulatum* IPLA 36007; lines S1 and S2, daidzin and daidzein standards, respectively. Arrows point to the position of daidzin and daidzein.

Among a large collection of intestinal bifidobacteria strains from human origin, *B. pseudocatenulatum* IPLA 36007 showed aglycone-releasing activity from isoflavone glycosides. Figure 1 shows the conversion of daidzin into daidzein after incubation with IPLA 36007 cells. Similar activity was detected using genistin as a substrate, which was transformed into genistein (data not shown). As this strain has already shown a bunch of key properties for its use as a probiotic, including among others good survival under conditions simulating those of the GIT, absence of undesirable enzyme activities and atypical antibiotic resistances, and ability to bind human intestinal epithelial cells [16], it was selected for whole genome sequencing in order to get insights on the molecular basis of its relevant phenotypic traits.

The general features of the *B. pseudocatenulatum* IPLA 36007 genome are summarized in Table 1. The draft genome sequence of IPLA 36007 included 23 contigs varying in size from 203 to 548,016 bp. According to the NCBI Prokaryotic Genome Annotation Pipeline the genome harbors 1,851 genes, of which 1,769 are coding sequences (CDS) and 22 are pseudogenes. Additionally, 60 predicted RNA genes were identified, including five copies of identical 16S, 23S and 5S rRNA genes, a non-coding RNA molecule (ncRNA), and 54 genes coding for tRNAs. The RAST server classified the CDS into 26 classes and 255 subsystems (sets of related functional roles).

**Table 1:** **Key features of the *B. pseudocatenulatum *** **IPLA 36007 genome**

**Characteristic**	**Figures**	**Observations**
Size of the genome	2,328,179 bp^a^	
G + C content	56.4%	
Contigs	23	(from 203 to 548,016 bp long)
Open reading frames (ORFs)	1851	
Coding sequences (CDS)	1769	
Pseudogenes	22	
RNA genes	60	5 rRNA operons, 54 tRNA, 1 ncRNA
Plasmidic genes	0	Plasmid free strain
Integrated phages	1	76 CDS
CRISPR-Cas system	1	22 repeats, 21 spacers, 8 CDS
Glycosyl-hydrolases and glycosyl transferases	>50	
β-glucosidases	5	5 glycosyl hydrolases family_3, 1 glycosyl hydrolase family_1

^a^bp, base pair; ncRNA, non-coding RNA.

This strain was demonstrated plasmid-free (data not shown), and, in agreement, no plasmid-associated genes were found. However, one integrated phage of around 43.5 kbp was recorded. The lysogenic phage region consisted of 76 CDS, and included a gene encoding a retron-type RNA-directed DNA polymerase typical of group II introns [17]. Phage-related sequences have been described in the genome of 22 strains of different bifidobacteria species, but only fragmentary information exists with regard to their functionality [18]. As in other bacteria, bifidobacterial prophages have been shown to possess classical modular genomic organization in which the DNA lysogeny module and the DNA packaging region are the most highly conserved.

**Figure 2:**
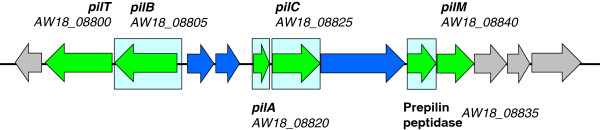
**Locus of the type IV fimbriae in *B. pseudocatenulatum *IPLA 36007.** In green, hortologous genes to those in other fimbriae/pili clusters. In blue, hypothetical genes belonging to the cluster. In grey, flanking CDS not related to the cluster. Pale blue background boxes indicate functionally coupled genes sharing conserved relative positions in the genome of at least four other species.

A cluster of nine genes capable of encoding type IV fimbriae or pili was identified. This contained, among others, orthologous genes for *pilA, pilB, pilC, pilM,* and *pilT* (Figure 2). Operons encoding type IV fimbriae have also been found in the genome of other sequenced *B. pseudocatenulatum* strains, in other bifidobacterial species, and in some environmental actinobacteria. Such pili have been well studied in Gram-negative bacteria such as *Neisseria gonorrhoeae*, *Pseudomonas aeruginosa*, *Pseudomonas stutzeri*, *Moraxella bovis* and *Dichelobacter nodosus*[19]. In these pathogenic bacteria, they mediate attachment and adherence to epithelial cells, twitching motility, gliding motility, cell agglutination, and biofilm and fruiting body formation [19]. In addition, they act as receptors for bacteriophages, and are required for extracellular protein secretion and natural transformation [19]. The expression and functionality of the type IV fimbriae genes in *B. pseudocatenulatum* IPLA 36007 has yet to be demonstrated.

**Figure 3:**
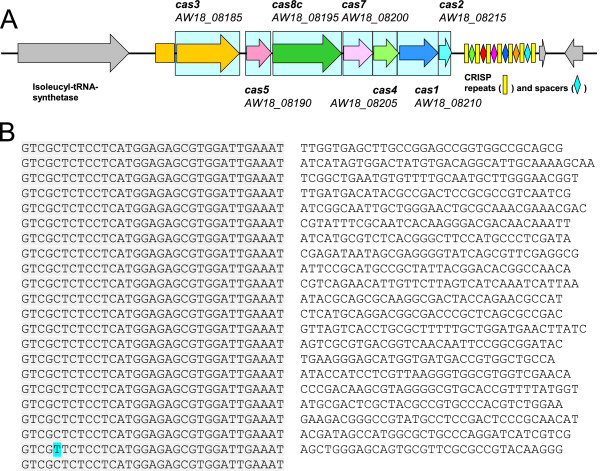
**Diagram of the CRISPR-Cas locus of *B. pseudocatenulatum *IPLA 36007. Panel A**: The type I CRISPR–Cas systems seem to target DNA. Orthologous genes are color coded following protein family relationships as suggested by Makarova et al. [20]. Cas3 is a large protein with separate helicase and DNase activities. Either Cas5 or Cas7 possess RNase activity. CRISP repeats and spacers are indicated by rectangles and rhombs. In grey, bounding CDS not related to the CRISPR-Cas system. **Panel B**: Twenty two identical 33 base pair (bp) repeats (except for repeat 21, which harbors a single bp change) are separated by unrelated 32 to 37 bp spacers. Pale blue background boxes indicate functionally coupled genes sharing conserved relative positions in the genome of at least four other species.

A locus encoding a CRISPR-Cas (clustered regularly interspaced short palindromic repeats-CRISPR-associated proteins) system was identified (Figure 3). This locus contained 22 identical repeats of 33 bp (except for one repeat carrying a single C-T transition at position five) and 21 spacers ranging from 32 to 37 bp (Figure 3B). The repeats (5′-GTCGCTCTCCTCATGGAGAGCGTGGATTGAAAT-3′) were preceded by eight CRISPR-associated (*cas*) genes (Figure 3A). Spacers showed no significant homology to DNA sequences on databases. Gene content and gene order analysis showed the CRISPR-Cas system of IPLA 36007 to belong to type I-C [20]. CRISPR-Cas systems provide defenses against foreign nucleic acids derived from bacteriophages, plasmids and other sources. These systems target and digest foreign DNA in an RNA-dependent, sequence-specific manner, and are also adaptive, providing protection against previously encountered exogenous elements [19]. Physiological roles for CRISPR-*cas* systems other than in defense against foreign DNA are slowly being uncovered [21].

**Figure 4:**
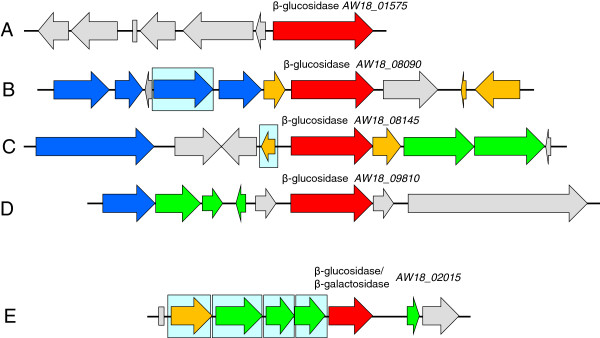
**Genetic organization around the putative β-glucosidase-encoding genes.** Five genes encoding glycosyl hydrosilases of family 3 **(A through D)** and a gene encoding a gycosyl hydrolase of family 1 **(E)** were identified in the *B. pseudocatenulatum* IPLA 36007 genome. Color code: glycosidase genes are in red; in green, genes involved in transport; in blue, genes involved in carbohydrate(s) metabolism; in brown, genes encoding regulator proteins; in gray, genes belonging to a distinct RAST subsystem; boxes represent genes encoding tRNA and 5S rRNA molecules. Pale blue background boxes indicate functionally coupled genes sharing conserved relative positions in the genome of at least four other species.

Bifidobacteria utilize a wide range of carbohydrates that escape digestion in the upper parts of the intestine, many of which are plant derived oligo- and polysaccharides [22]. The genome of *B. pseudocatenulatum* IPLA 36007 contains a vast array of glycosyl transferases and glycosyl hydrolases CDS (52 genes), including genes that code for xylanases (4), pullulanases (3), amylomaltases (2) α-amylase (1) and maltodextrin glucosidase (1). It also harbors genes encoding α- (5) and β- (9) galactosidases, α- (1) and β- (7) xylosidases, α- (1) and β- (4) glucosidases, α-arabinofuranosidases (4), β- mannosidases (2), and α-rhamnosidases (1). Although belonging to the glycosyl hydrolase family 3, all four β-glucosidases (EC 3.2.1.21) shared very limited amino acid identity (26.2%) (Figure 4, A through D). Multiblast analysis of the β-glucosidases found in the IPLA 36007 genome and those present in bifidobacterial genomes suggested that homologous enzymes are present in bifidobacteria strains belonging to the *Bifidobacterium adolescentis* group (*B. adolescentis, B. dentium, B. angulatum, B. catenulatum*, etc.) (amino acid identity ranging from 80 to 98%). The homology to glycosyl hydrolases from other groups of bifidobacteria was much lower (less than 70% amino acid identity) (Additional file 1: Figure S1). Indeed, the similarity of the deduced β-glucosidases from IPLA 36007 to that of *B. animalis* subps. *lactis* SH5, which have been proved to act on soy isoflavone glycosides [14], was found to be marginal (23% amino acid identity). An additional gene encoding a putative glycoside hydrolase belonging to family 1 (EC 3.2.1.23) was detected; this might also have β-glucosidase activity (Figure 4, E). Characterization of these genes and their encoded glycosyl hydrolase enzymes would allow those acting on soy isoflavone glycosides to be identified. However, hydrolysis of isoflavone glycosides some others glycosyl hydrolases of those detected cannot be discarded.

The availability of the genome of the *B. pseudocatenulatum* IPLA 36007 strain should allow the enzymes involved in the release of soy isoflavone aglycones from isoflavone glycosides to be known. This is essential for the rational use of IPLA 36007 as a probiotic in functional foods. The ability of IPLA 36007 to colonize the GIT could be exploited to deliver the aglycone-releasing activity straightway into the human intestine. Comparison of sequences from different sequenced strains would provide greater insights into the genetic variation within this species. It will further allow the core genome and pangenome of *B. pseudocatenulatum* to be identified, while contributing towards defining the gene set required to be competitive in the human GIT.

## Competing interests

The authors declare that they have no competing interests.

## Authors’ contributions

BM and SD designed the study. AA, ABF and LG performed the experiments. AA, SD, BM, GEF and ST analyzed the data. BM and SD wrote the manuscript. AA, GEF and ST checked and edited the manuscript. All authors read and approved the final manuscript.

## Supplementary Material

Additional file 1: Figure S1. Multiblast analysis of putative β-glucosidases from *Bifidobacterium pseudocatenulatum* IPLA 36007 to those on the genomes of other bifidobacteria in the databases. Click here for file
