# Supplementary material for: The genome of Bifidobacterium pseudocatenulatum IPLA 36007, a human intestinal strain with isoflavone-activation activity
Source: Gut Pathog. 2014 Jul 23;6:31. doi: 10.1186/1757-4749-6-31 (PMC4121622; doi:10.1186/1757-4749-6-31)
Supplement: Additional file 1: Figure S1. — Multiblast analysis of putative β-glucosidases from Bifidobacterium pseudocatenulatum IPLA 36007 to those on the genomes of other bifidobacteria in the databases. [file 1757-4749-6-31-S1.pdf]

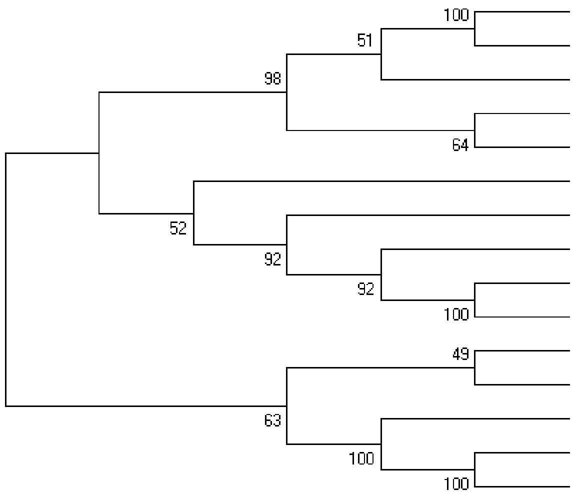

Strain	BG 1599	BG 1608	BG 1944	BG 305	BG 385
B.catenulatum str. DSM 16992	93,15	98,22	91,19	85,75	
B.pseudocatenulatum DSM 20438 JCM 1200	93,02	99,82	99,47	99,72	87,84
B.angulatum	80,84	82,31			97,96
B.adolescentis ATCC 15703	93,19	85,66	84,93	85,12	92,26
B.dentium Bd1	83,35	85,04	82,94	85,17	
B.bifidum					
B.subtile			72,84	76,98	
B.breve UCC2003 complete gen[illegible]	78,23	77,81	82,94	71,52	71,96
B.longum subsp. infantis ATCC 15697					
B.longum subsp. longum JCM 1217	78,95	77,99			
B.asteroides PRL2011	66,18	66,97	66,64		
B.thermophilum RBL67 strain RE	78,07				
B.gallicum DSM 20093					
B.animalis subsp. animalis ATCC 25527	72,41				
B. animalis subsp. lactis BLC1	72,82	68,63			

Supplementary Figure 1. Multiblast analysis of putative β-glucosidases from *Bifidobacterium pseudocatenulatum* IPLA 36007 to those on the genomes of other bifidobacteria in the databases.
